# Supplementary material for: When and where? Day-night alterations in wild boar space use captured by a generalized additive mixed model
Source: PeerJ. 2024 Jun 12;12:e17390. doi: 10.7717/peerj.17390 (PMC11179635; doi:10.7717/peerj.17390)
Supplement: Supplemental Information 1 [file peerj-12-17390-s001.docx]

**When and Where? Day-night Alterations in Wild Boar Space Use Captured by a Generalized Additive Model.**

Bollen Martijn, Casaer Jim, Neyens Thomas and Beenaerts Natalie

Supplementary file S1: Hunting effort per management zone.

**Table S1.** Number of hunting records, the total effort in hours and attempted shots registered during the summer (April – September) for each of the management zones in Meerdaal.

| Zone | Summer | No. records  (% of annual total) | Effort in hours  (% of annual total) | No. shots  (% of annual total) |
| --- | --- | --- | --- | --- |
| Core | 2018 | 1 (0.68) | 12 (2.51) | 0 (0.00) |
|  | 2019 | 2 (1.02) | 5 (1.03) | 0 (0.00) |
|  | 2020 | 1 (0.48) | 3 (0.51) | 0 (0.00) |
|  | 2021 | 0 (0.00) | 0 (0.00) | 0 (0.00) |
| Winter  Hunting | 2018 | 2 (1.37) | 8 (1.67) | 0 (0.00) |
|  | 2019 | 3 (1.53) | 5 (1.03) | 1 (3.12) |
|  | 2020 | 2 (0.96) | 4 (0.67) | 0 (0.00) |
|  | 2021 | 1 (0.65) | 1 (0.20) | 1 (3.57) |
| Year-round  Hunting | 2018 | 143 (97.95) | 459 (95.82) | 20 (100) |
|  | 2019 | 191 (97.45) | 477 (97.95) | 31 (96.88) |
|  | 2020 | 205 (98.56) | 586 (98.82) | 29 (100) |
|  | 2021 | 153 (99.35) | 492 (99.8) | 27 (96.43) |
