# Supplementary material for: When and where? Day-night alterations in wild boar space use captured by a generalized additive mixed model
Source: PeerJ. 2024 Jun 12;12:e17390. doi: 10.7717/peerj.17390 (PMC11179635; doi:10.7717/peerj.17390)
Supplement: Supplemental Information 2 [file peerj-12-17390-s002.docx]

**When and Where? Day-night Alterations in Wild Boar Space Use Captured by a Generalized Additive Model.**

Bollen Martijn, Casaer Jim, Neyens Thomas and Beenaerts Natalie

Supplementary file S2: Information reduced GAM to model diel space use for wild boar

In the main paper we presented a GAM that models wild boar encounter rates $\lambda_{it}$ from counts $y_{ijt}$ retrieved from cameras $i = 1, 2, \ldots, R$ with coordinates $\{lon(i), lat(i)\}$, on survey days $j = 1,2, \ldots, J_{i}$ and solar hours $t = 0, \frac{2\pi}{24}, ..., 2\pi$. Here, we present a simpler model using inputs $y_{it}$ obtained by summation of counts $y_{ijt}$ across survey days $J_{i}$ on which the $i$^th^ camera was active and that increases the percentage of non-zero counts. This model is expressed as:

$$y_{it}\sim NegBin\left( \lambda_{it},\theta\right),$$

$log(\lambda_{it})=\beta_{0}+log(J_{i})+f_{1}(t)+f_{3}\left( t,lon\left( i \right),lat\left( i \right) \right)$*,*

with the total number of survey days $J_{i}$ at the $i$^th^ camera as an offset term, such that the encounter rates $\lambda_{it}$ represent the expected number of wild boars captured during solar hour $t$ of any given day (instead of the expectation across all days). Note that this model still permits the modelling of smooth curves for solar hour, *i.e.,* $f_{1}(t)$ and for the combination of solar hour and spatial location, *i.e.,* $f_{3}\left( t,lon\left( i \right),lat\left( i \right) \right)$, but not for week of the year $f_{2}(week(j))$. Additionally, it is not possible anymore to specify random effects for survey days $j$, *i.e.,* $\beta_{0,j}$. Hereafter, we provide the residual plots and spatiotemporal predictions from this model showing that the goodness-of-fit improves under this model, while the diel space use inferred from it is qualitatively the same as that estimated from the extended model of the main paper.


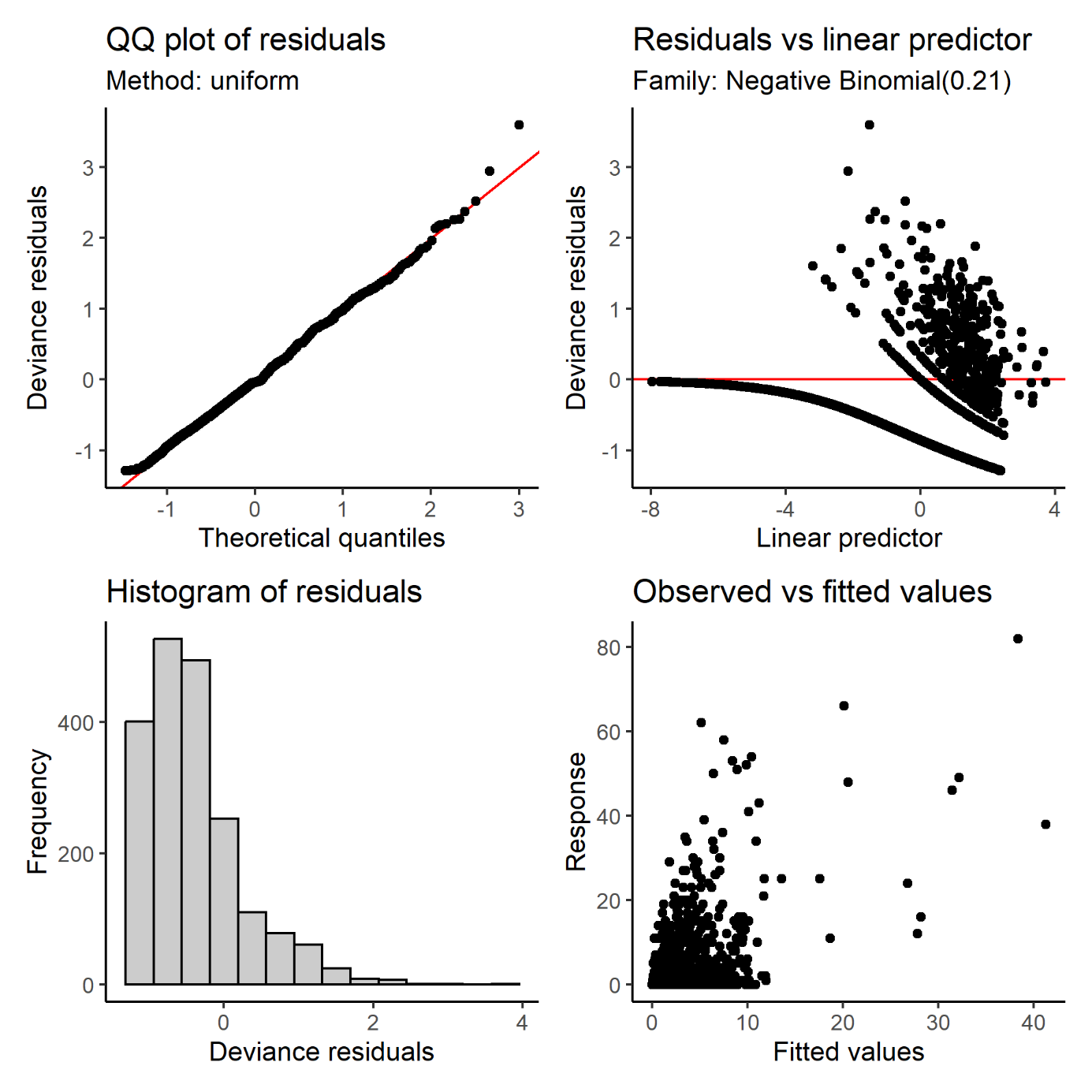


**Figure S2.1.** Residual plots for a negative binomial GAM modelling wild boar diel space use from counts aggregated across survey days.

*
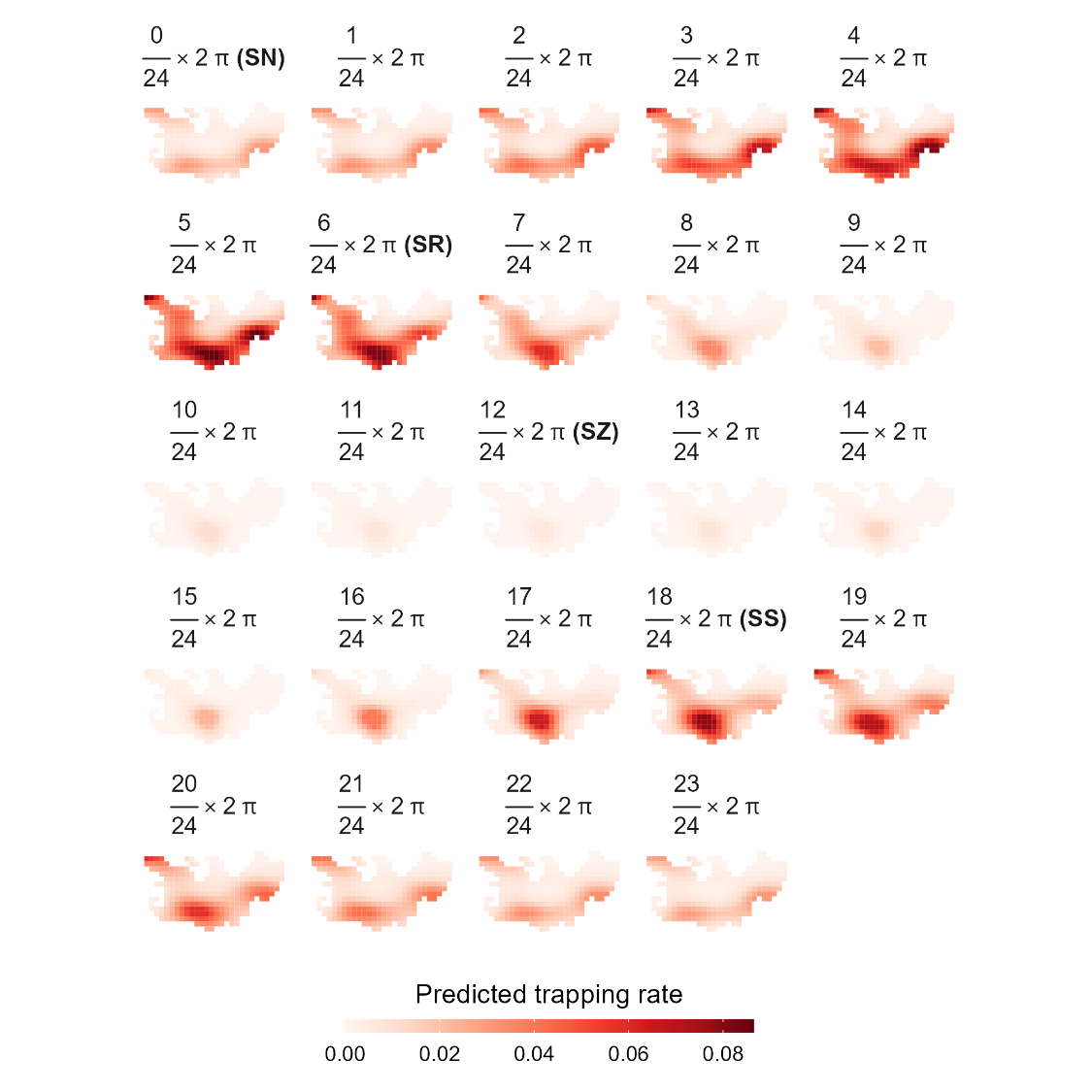
*

**Figure S2.2.** Predicted spatiotemporal variation in wild boar trapping rates from the reduced model across 24 solar hours. SN: sun nadir, SR: sunrise, SZ: sun zenith, SS: sunset.


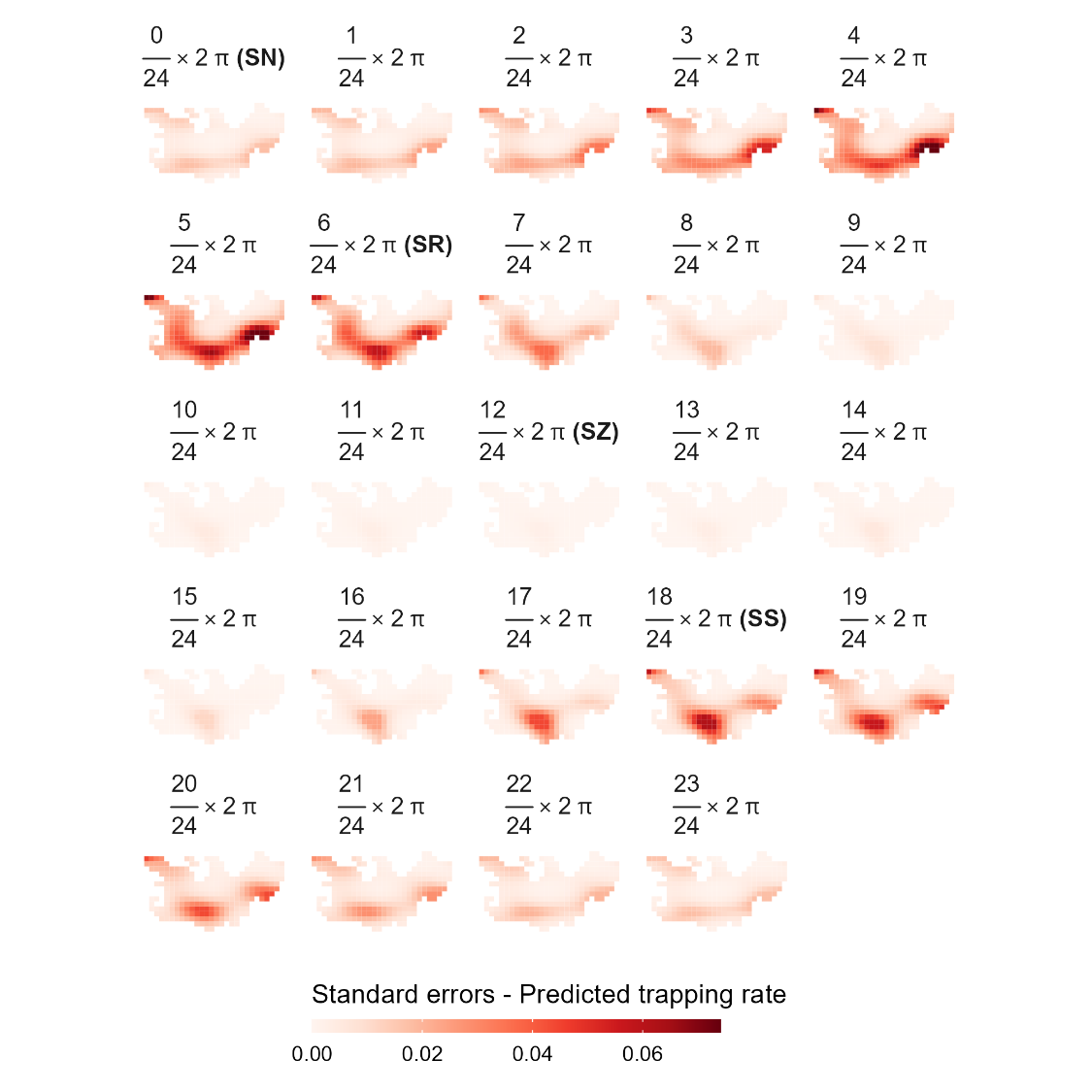


**Figure S2.3.** Standard errors in predicted spatiotemporal variation in wild boar trapping rates from the reduced model across 24 solar hours. SN: sun nadir, SR: sunrise, SZ: sun zenith, SS: sunset.
