# Supplementary material for: When and where? Day-night alterations in wild boar space use captured by a generalized additive mixed model
Source: PeerJ. 2024 Jun 12;12:e17390. doi: 10.7717/peerj.17390 (PMC11179635; doi:10.7717/peerj.17390)
Supplement: Supplemental Information 3 [file peerj-12-17390-s003.docx]

**When and Where? Day-night Alterations in Wild Boar Space Use Captured by a Generalized Additive Model.**

Bollen Martijn, Casaer Jim, Neyens Thomas and Beenaerts Natalie

Supplementary file S3: Residual plots for the GAMs presented in the main paper


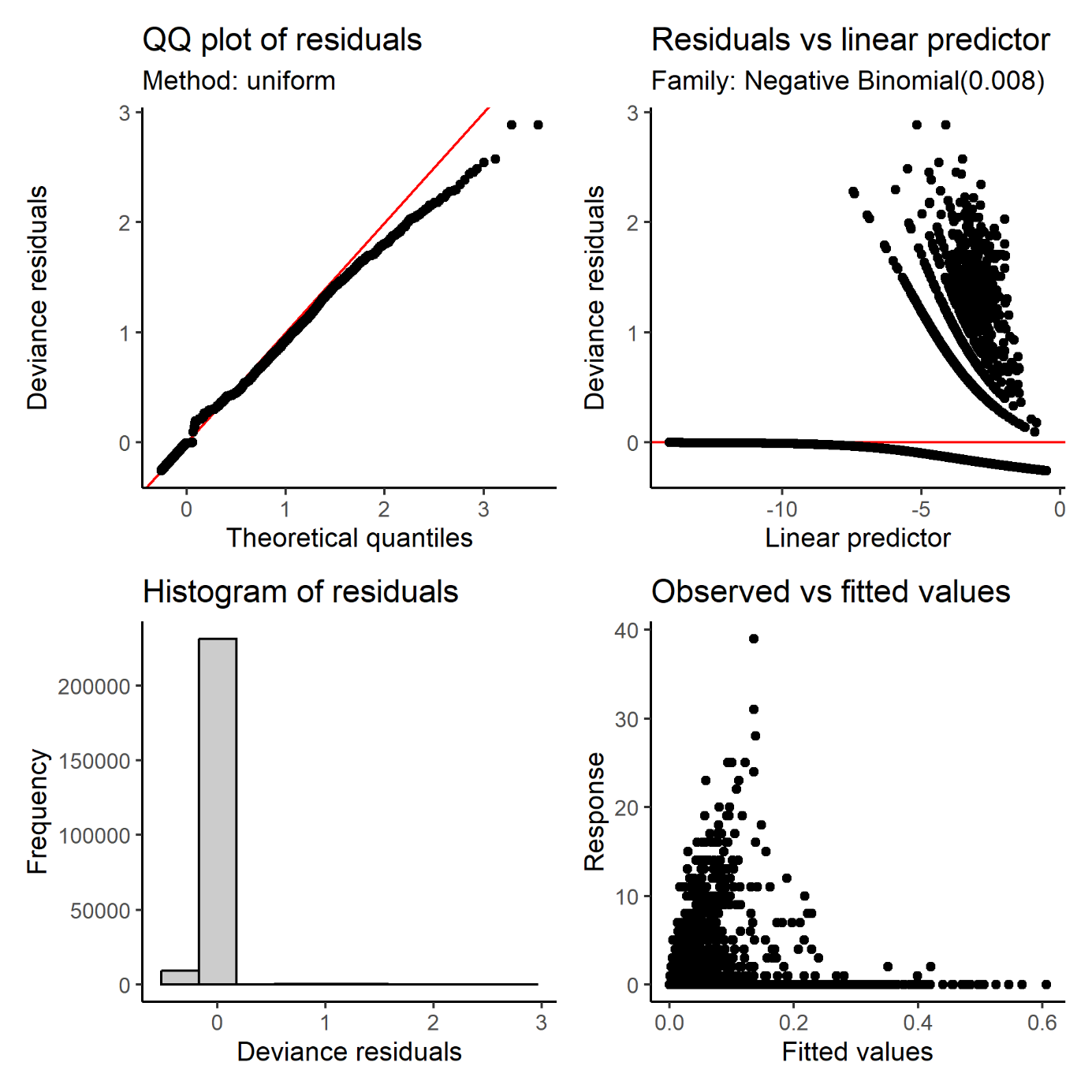


**Figure S3.1.** Residual plots for a negative binomial GAM modelling wild boar diel space use.


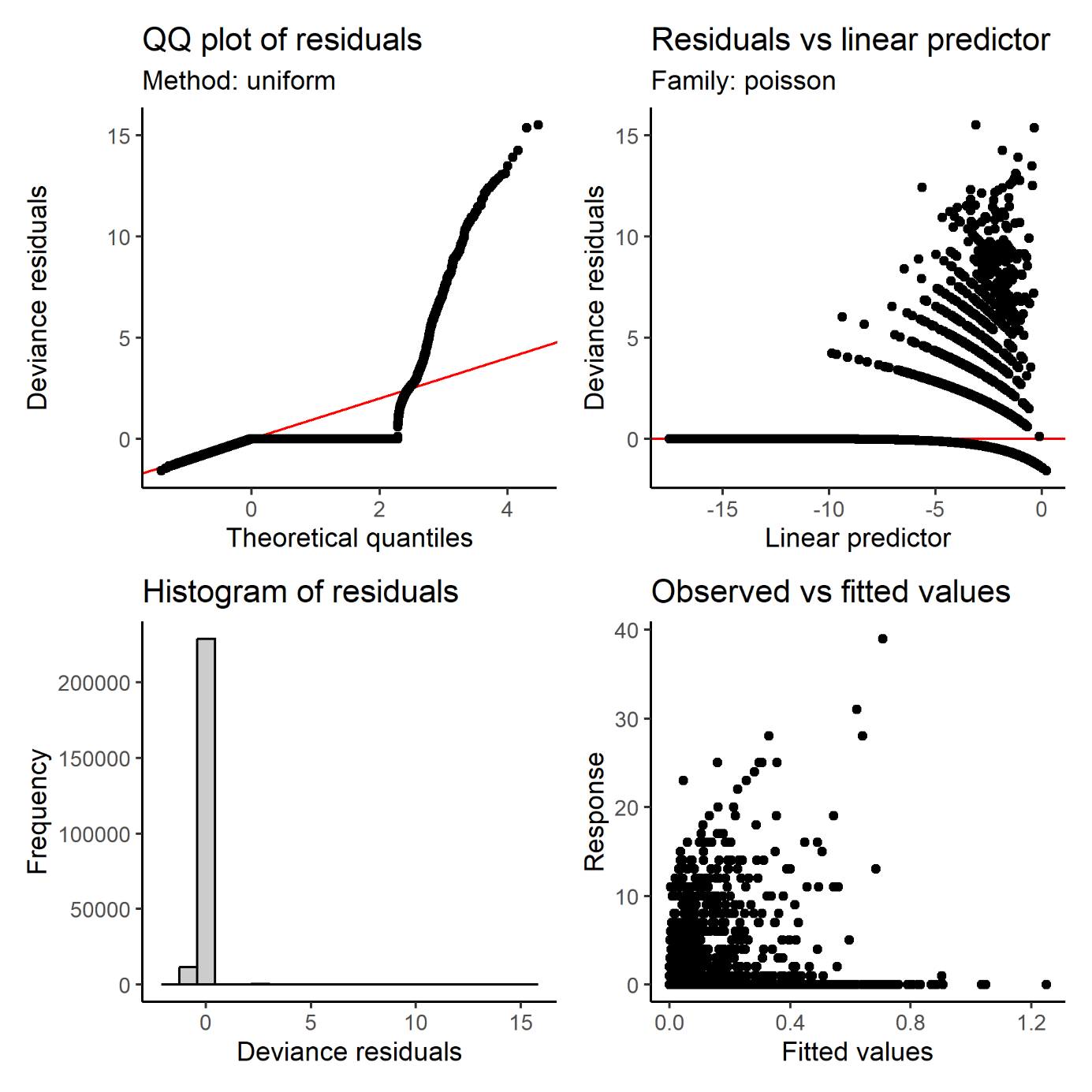


**Figure S3.2.** Residual plots for a Poisson GAM modelling hunter encounters across space and solar time bins.


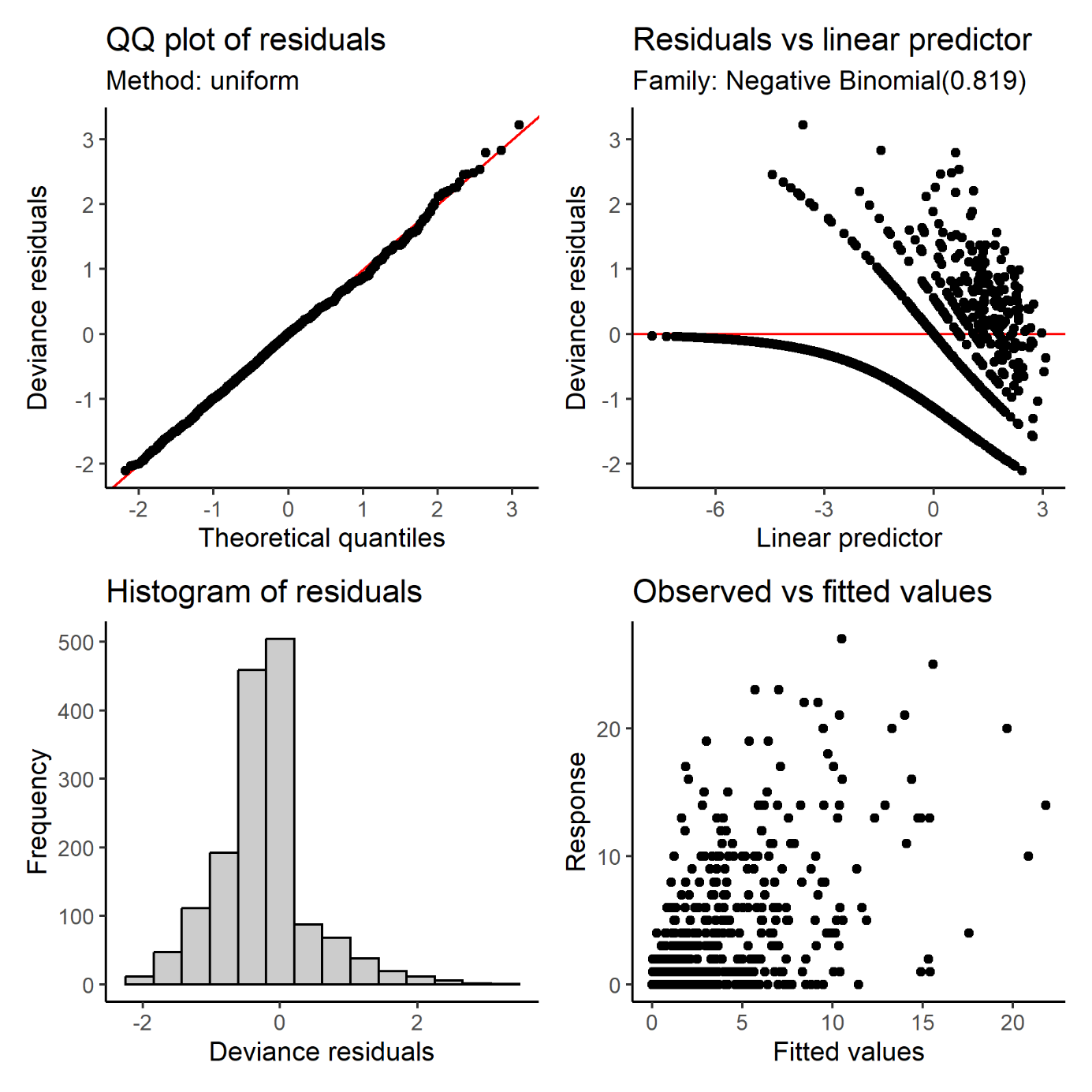


**Figure S3.3.** Residual plots for a negative binomial GAM modelling hunter encounters across space and solar time bins.


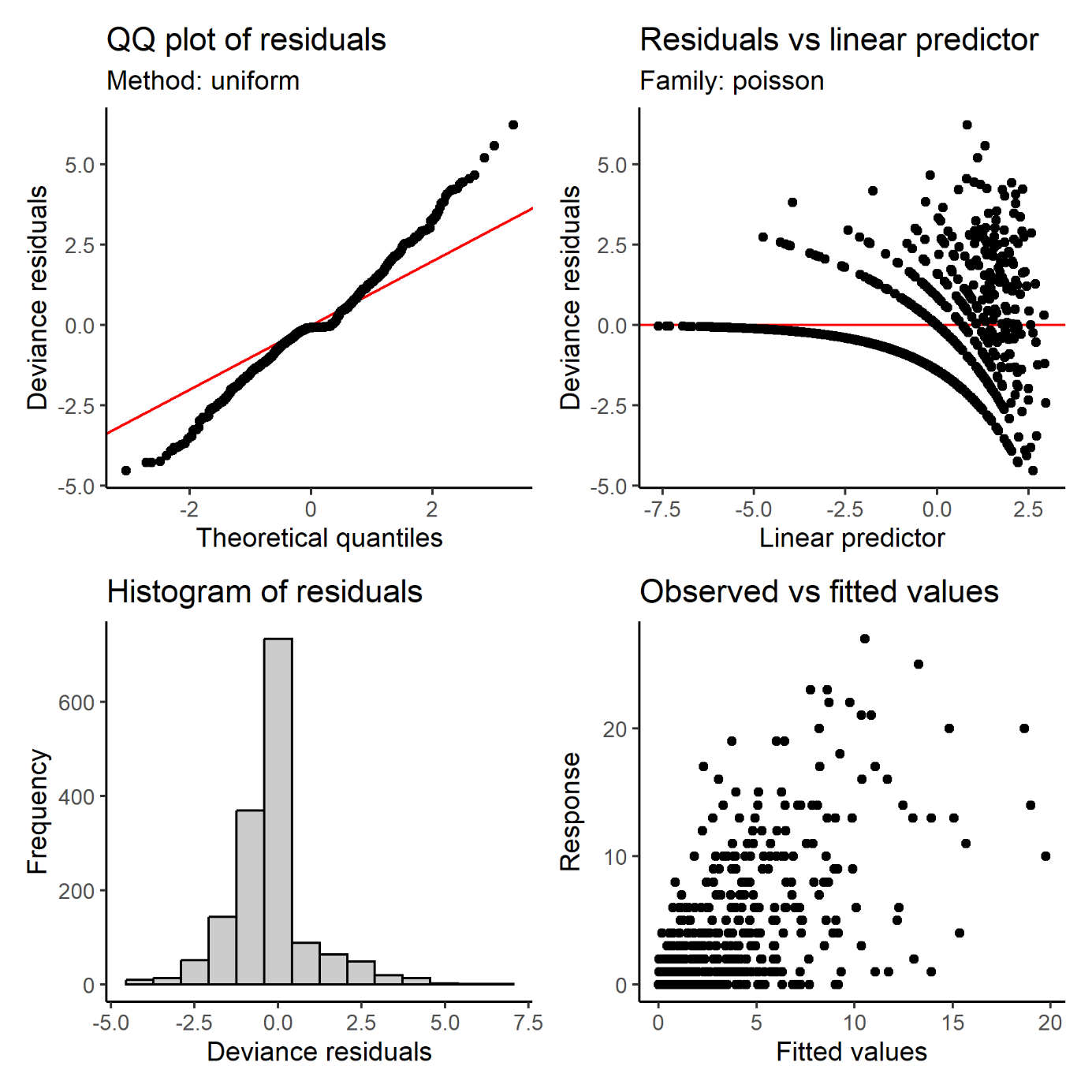


**Figure S3.4.** Residual plots for a Poisson GAM modelling wild boar encounters on days with hunting across space and solar time bins.
