# Supplementary material for: When and where? Day-night alterations in wild boar space use captured by a generalized additive mixed model
Source: PeerJ. 2024 Jun 12;12:e17390. doi: 10.7717/peerj.17390 (PMC11179635; doi:10.7717/peerj.17390)
Supplement: Supplemental Information 4 [file peerj-12-17390-s004.docx]

**When and Where? Day-night Alterations in Wild Boar Space Use Captured by a Generalized Additive Model.**

Bollen Martijn, Casaer Jim, Neyens Thomas and Beenaerts Natalie

Supplementary file S4: Spatiotemporal predictions from the GAMs presented in the main paper


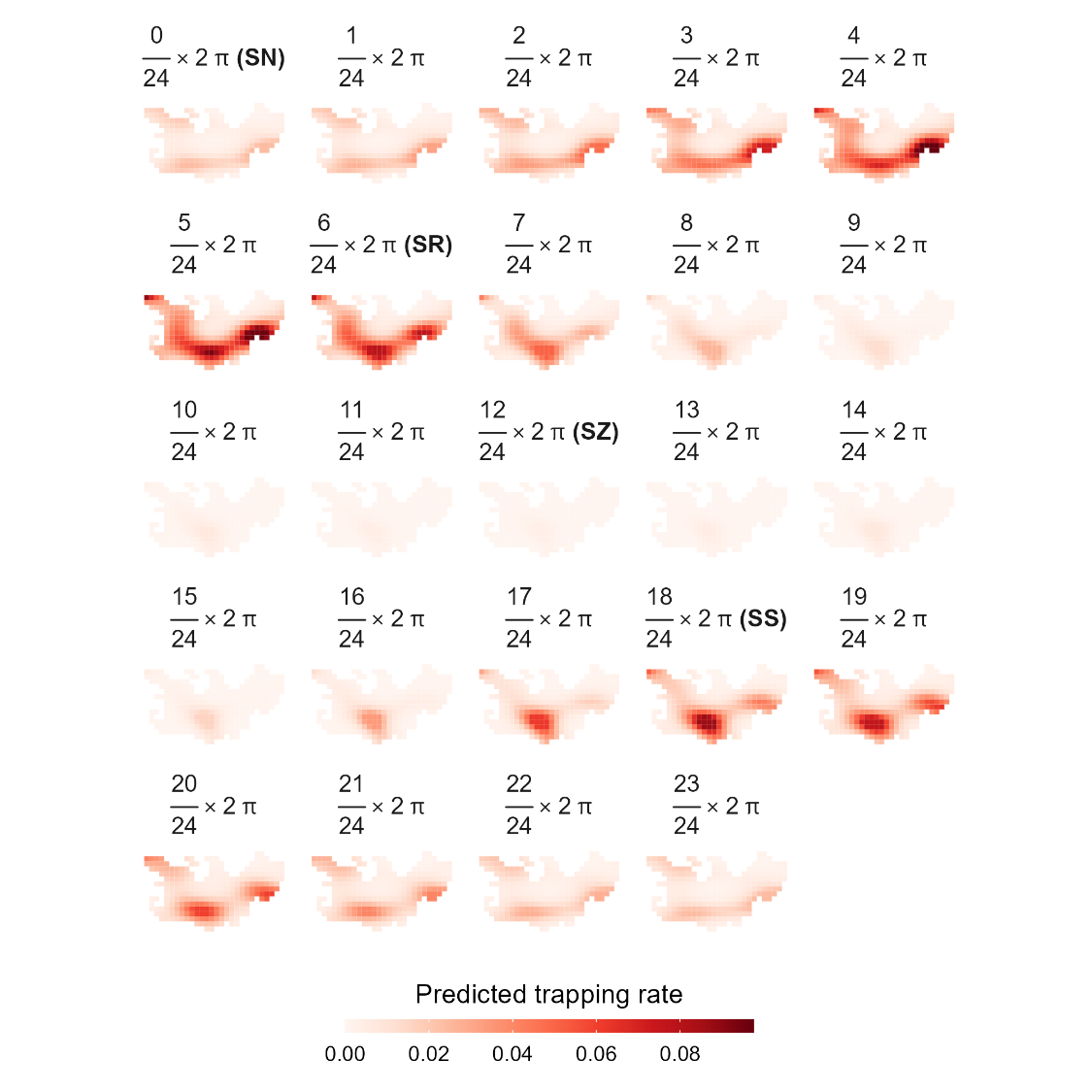


**Figure S4.1.** Predicted spatiotemporal variation in wild boar trapping rates across 24 solar hours. SN: sun nadir, SR: sunrise, SZ: sun zenith, SS: sunset.


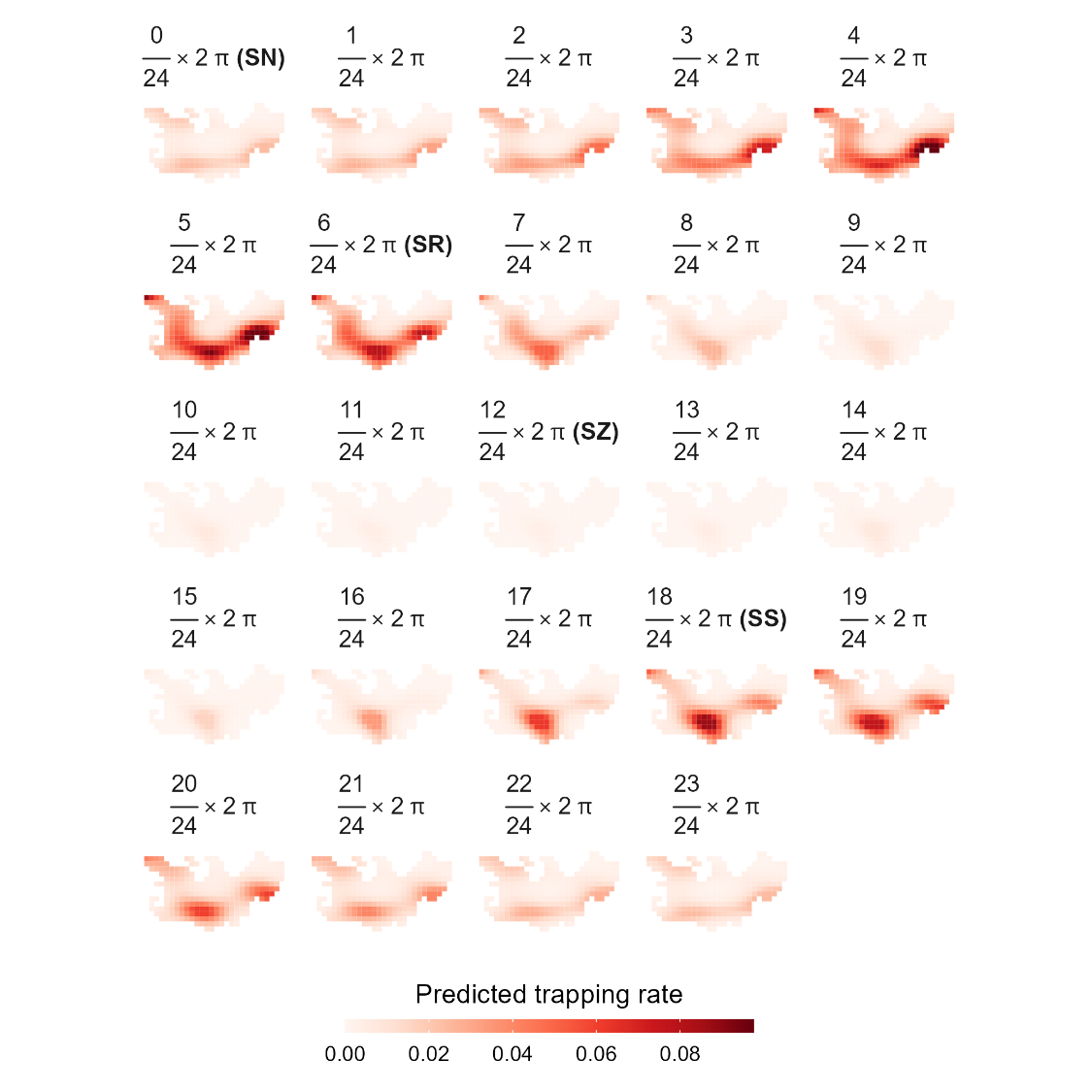


**Figure S4.2.** Standard errors in predicted spatiotemporal variation in wild boar trapping rates across 24 solar hours. SN: sun nadir, SR: sunrise, SZ: sun zenith, SS: sunset.


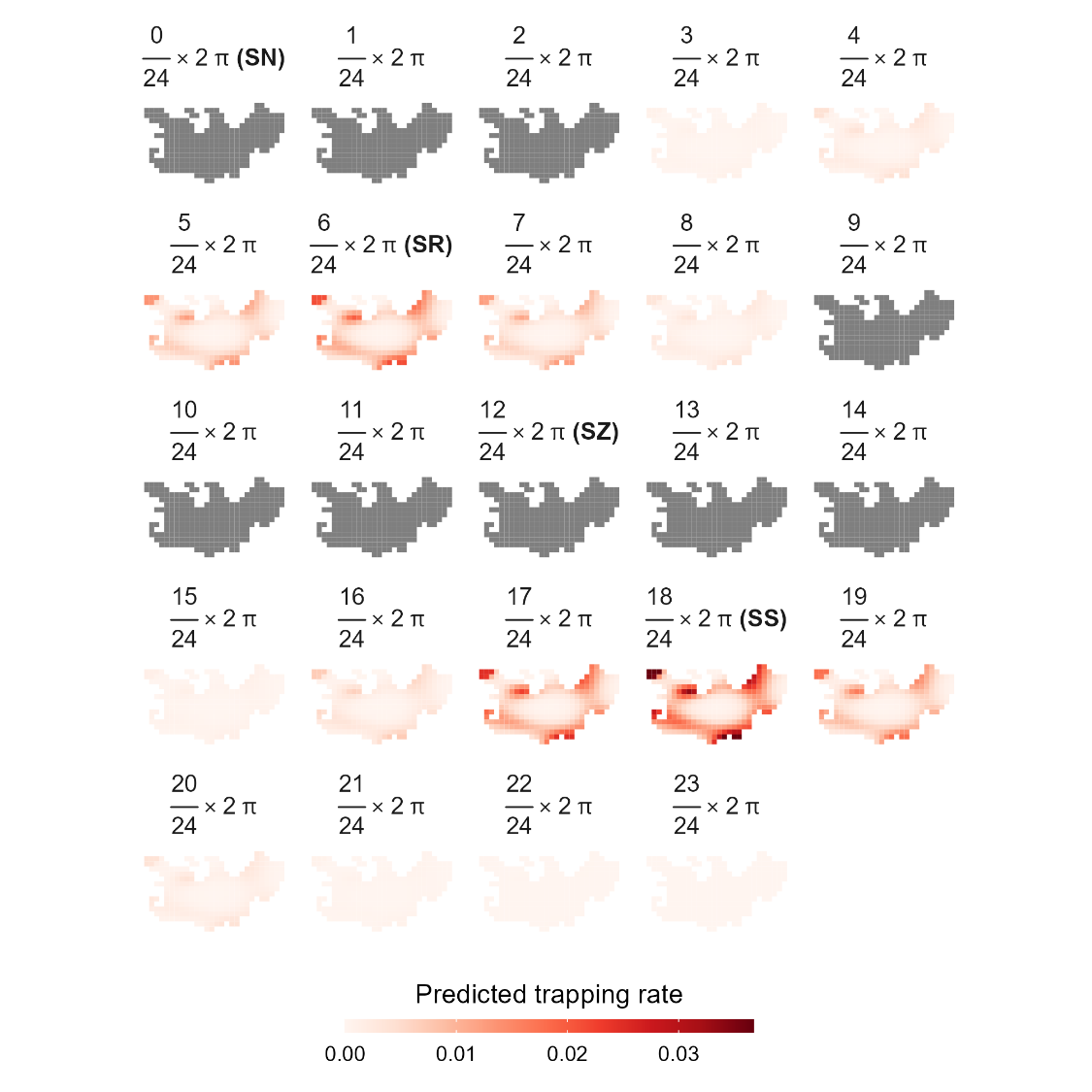


**Figure S4.3.** Predicted spatiotemporal variation in hunting pressure across 24 solar hours. SN: sun nadir, SR: sunrise, SZ: sun zenith, SS: sunset. The grey maps indicate solar hours at which there were too few records to estimate hunting pressure (grey maps).


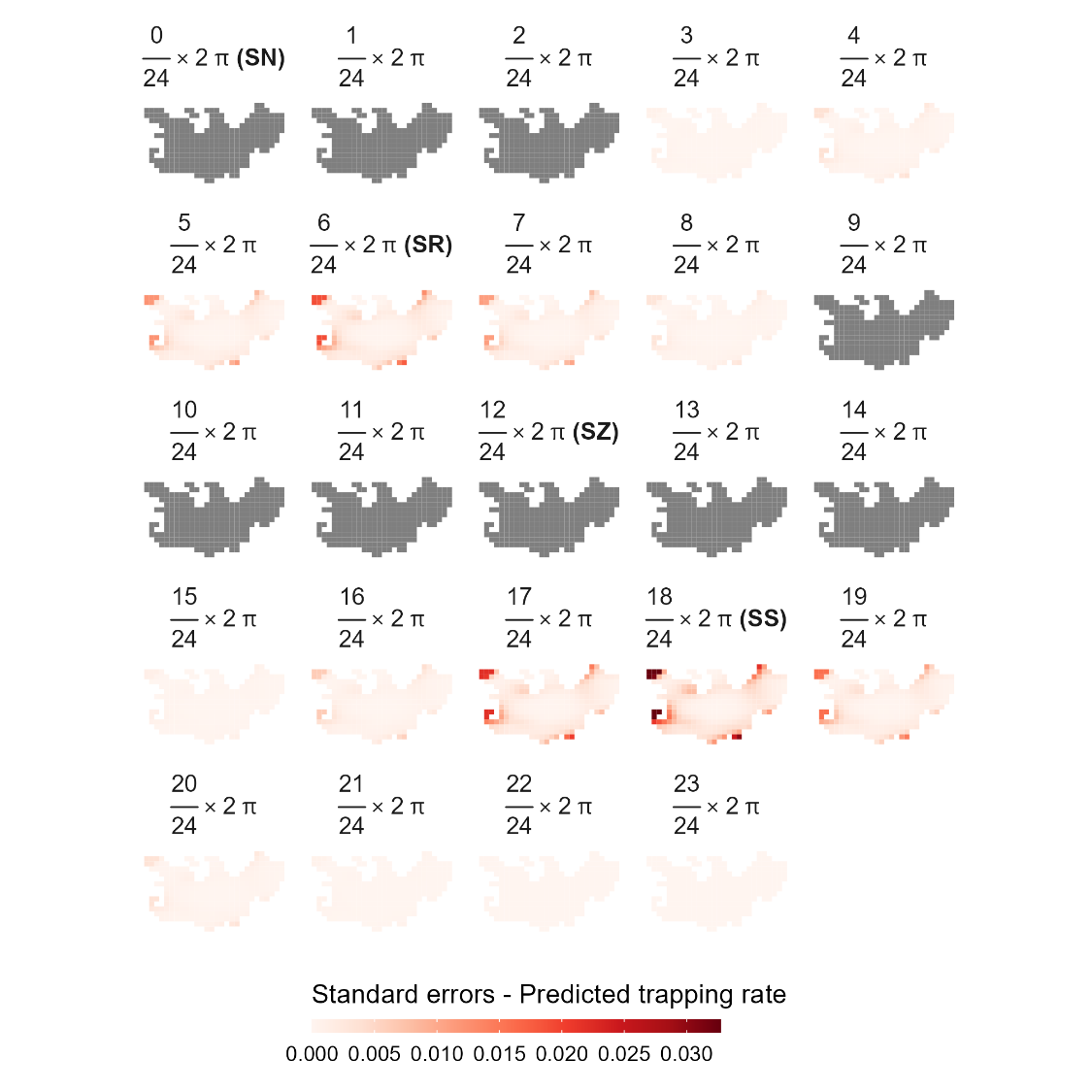


**Figure S4.4.** Standard errors predicted spatiotemporal variation in hunting pressure across 24 solar hours. SN: sun nadir, SR: sunrise, SZ: sun zenith, SS: sunset. The grey maps indicate solar hours at which there were too few records to estimate hunting pressure (grey maps).
